# Semiparametric Trend Analysis for Stratified Recurrent Gap Times Under Weak Comparability Constraint

**DOI:** 10.1007/s12561-023-09376-8

**Published:** 2023-06-03

**Authors:** Peng Liu1, Yijian Huang, Kwun Chuen Gary Chan, Ying Qing Chen

**Affiliations:** 1 School of Mathematics, Statistics and Actuarial Science, University of Kent, Canterbury, Kent CT2 7FS, UK; 2 Department of Biostatistics, Rollins School of Public Health, Emory University, Atlanta, GA 30322, USA; 3 Department of Biostatistics, University of Washington, Seattle, WA 98195, USA; 4 Stanford Prevention Research Center, Stanford University, Palo Alto, CA 94305, USA

**Keywords:** Accelerated failure time model, Comparability, Gap time, Rank regression, Recurrent event data

## Abstract

Recurrent event data are frequently encountered in many longitudinal studies where each individual may experience more than one event. Wang and Chen (Biometrics 56(3):789–794, 2000) proposed a comparability constraint to estimate the time trend for the gap times, where the gap time pairs that satisfy the constraint have the same conditional distribution. However, the comparable paired gap times are also independent. Therefore, the comparable gap time pairs will be subject to a stronger constraint than needed for the estimation. Thus their procedure is subject to information loss. Under the accelerated failure time model, we propose a new comparability constraint that can overcome the drawback mentioned above. The gap time pairs being selected by the proposed comparability constraint will still have the same distribution, but they do not need to be independent of each other. We showed that the proposed comparability constraint will utilize more gap time data pairs than the strong comparability. And we showed via various simulation studies that the variance will be smaller than Wang and Chen’s (2000) estimator. We apply the proposed method to the HIV Prevention Trial Network 052 study.

## 1 Introduction

Recurrent event data are frequently encountered in many longitudinal studies when a particular event of interest repeatedly occurs for a subject. Examples include the cancer recurrence, women’s menstrual cycles, and machinery breakdown. Assume there are n subjects in a study who have experienced an initial event (e.g. cancer occurrence). Let i=1,…,n index the subjects and j=0,1,… index the recurrent events for a given subject, where j=0 denotes the initial event. For subject i, let $T_{ij}$ be the gap time, which is the time between (j-1) th and jth events, let $C_{i}$ be the time between the beginning of the study and the end of follow-up, then:

(1) $$\sum_{j=1}^{m_{i}-1}\;T_{ij}\leq C_{i},\sum_{j=1}^{m_{i}}\;T_{ij}>C_{i,}$$

where $m_{i}$ is the number of observed gap times. When $m_{i}=1,T_{i1}$ is being censored at $C_{i}$, then we define $\sum_{j=1}^{m_{i}-1}T_{ij}=0$. Otherwise, if $m_{i}>1$, then the first $m_{i}-1$ gap times are complete and the last one is being censored at $T_{i,m_{i}}^{+}=C_{i}-\sum_{j=1}^{m_{i}-1}T_{ij}$. The observed gap times consists of $\left\{T_{i1},\ldots ,T_{i,m_{i}-1},T_{i,m_{i}}^{+}\right\}$ for subject i. We assume the observed data are i.i.d. across the n subjects.

One particular research interest in studying the recurrent event data is the time trend analysis for the gap times [2–4]. The trend analysis is of scientific importance due to its application in measuring disease progression. For example, researchers are often interested in whether a treatment for a psychiatric patient will prolong the time for readmission of hospitalization, since frequently readmission means the treatment is not effective [5]. An explicit idea to study the time trend is to compare the length of different gap times according to chronological order. However, it is impossible to conduct a naive comparison, since the recurrent event data are subject to induced censoring [6], which means $T_{ij}s(j\geq 2)$ are subject to dependent censoring by $C_{i}-T_{i1}-\cdots -T_{i,j-1}$. [1] tackled the induced censoring issue by comparing the marginal distributions of different gap times, and proposed to study the time trend by conducting a hypothesis testing procedure. The null hypothesis states that there is no time trend, i.e., all the $T_{ij}s$ have the same marginal distribution within each subject i. The standard K-sample trend test can be applied if there is no censoring. However, in the presence of induced censoring, this approach will not work. One way to circumvent this issue is to introduce the comparability concept, where comparability means a further constraint to the different combinations of pairwise gap times within a subject, so that the gap time pairs that satisfy the comparability constraint can still be comparable (e.g. have the same conditional marginal distribution). Given subject i, for gap time pairs $\left(T_{ij},T_{ik}\right)\;(j<k)$, $\sum_{l=1}^{k}T_{il}\leq C_{i}$ ensures that both $T_{ij}$ and $T_{ik}$ will not be censored. Denote $S_{i,jk}=\sum_{l=1}^{k}T_{il}-\left(T_{ij}+T_{ik}\right)$, given $T_{i1},\ldots ,T_{ik}$ and $C_{i}$, in order to avoid $T_{ij}$ and $T_{ik}$ being censored, $T_{ij}+T_{ik}$ must not be greater than $C_{i}-S_{i,jk}$. According to [1]’s definition, $T_{ij}$ and $T_{ik}$ are comparable if $T_{ij}$ can be fitted into $T_{ik}$’s observation interval and vice versa. Here the observation intervals for $T_{ij}$ and $T_{ik}$ are $C_{i}-S_{i,jk}-T_{ij}$ and $C_{i}-S_{i,jk}-T_{ik}$, respectively. For further details of the rationale, please see Sect. 2 in [1]. In the absence of covariates, the comparability constraint in [1] is defined as:

(2) $$T_{ij}\;\leq C_{i}-S_{i,jk}-T_{ij,}\;T_{ik}\;\leq C_{i}-S_{i,jk}-T_{ik}.$$

If $T_{ij}$ and $T_{ik}$ satisfy the constraint (2), then they will be a comparable pair. It is worth mentioning that the comparability concept also appears in regression problems based on independent truncated observations, see for example [7–9], among others.

In the presence of censoring, [1] proposed the comparability constraint and construct a test statistic for the trend analysis

(3) $$U=\sum_{i=1}^{n}\;\sum_{j<k\leq m_{i}-1}\;\delta_{i,jk}\text{sgn}\left\{\left(T_{ik}-T_{ij}\right)\left(Z_{ik}-Z_{ij}\right)\right\},$$

where $Z_{ij}$ is a given trend measure for jth gap time of subject i, and $\delta_{i,jk}$ is the comparability constraint. One practical example of the trend measure is the dose level [?]. An assigned measure such that $Z_{ij}$ is increasing with j can also be used, such as $Z_{ij}=j$ [10]. Here $\delta_{i,jk}$ stands for the comparability constraint that is imposed on different gap time pairs $T_{ij}$ and $T_{ik}$ for subject i, if $T_{ij}$ and $T_{ik}$ is comparable, then we have $\delta_{i,jk}=1$, otherwise, define $\delta_{i,jk}=0$. Thus one can see that only comparable pairs are being selected and used in the hypothesis testing. Since the last observation $T_{i,m_{i}}^{+}$ is always biased due to intercept sampling problem [6], $T_{i,m_{i}}^{+}$ will be excluded from (3) as well as the following statistical analysis.

In the presence of covariates, [1] adapted the comparability constraint to the accelerated failure time model, and included the trend measure $Z_{ij}$ as one component of the covariates, thus the sign of the coefficient of the trend measure covariate can be used to determine the trend. As a result, one only need to estimate the trend via a parameter estimation procedure. However, as can be shown in Sect. 2.1 of this paper, under the comparability constraint proposed by [1], the comparable gap time pairs will not only have the same distribution, but also are independent. Therefore, [1]’s estimation procedure under the accelerated failure time model will be subject to information loss. We also conduct simulation studies and the results show that the comparability constraint in [1]’s paper only use a half of the data under moderate censoring, when the censoring is heavy, the information loss will become even worse (Tables 1 and 2 in Sect. 3).

In this paper, we propose an alternative comparability constraint under the same model as in [1]. We want to mention that compared with [1]’s estimation procedure, our estimation procedure employs the same assumptions as in their paper. More importantly, we proved that the comparable pairs under the new comparability constraint will have the same conditional distribution, but they are not conditional independent. Thus our method will be superior to [1]’s. Since our constraint is weaker, we name our method and [1]’s as the weak comparability and strong comparability, respectively. Our simulation results also show that the weak comparability can recruit more comparable gap time pairs, and the variance for our estimator will be smaller than the estimator under the strong comparability.

This paper is organized as follows: In Sect. 2, we introduce the concept of weak comparability, and show the asymptotic results. Section 3 presents the simulation results. The proposed method is applied to the HIV Prevention Trial Network 052 data in Sect. 4. The paper is finalized by discussion in Sect. 5.

## 2 Main Results

### 2.1 The Strong Comparability Under the Accelerated Failure Time Model

Given subject i, for jth gap time $\left(j=1,\ldots ,m_{i}-1\right)$, consider the following accelerated failure time model:

(4) $$\text{log}\;T_{ij}=\alpha_{i}+Z_{ij}^{⊤}\beta +e_{ij},$$

where $\alpha_{i}$ is a random intercept, $Z_{ij}$ is a p×1 vector of covariates whose values vary within subject i with respect to different gap times, and one component of $Z_{ij}$ is the trend measure, β is a p×1 vector of parameters. For subject i, conditioning on $\alpha_{i}$ and $Z_{ij}$, the error terms $e_{ij},j=1,\ldots ,m_{i}-1$ are independent from each other and have a common distribution $G_{i}$ with mean zero. If p=1 and $Z_{ij}$ is the trend measure, then model (4) provides a direct interpretation of time trend for gap times: β=0 means no trend, β>0/β<0 means that $T_{ij},j=1,\ldots ,m_{i}-1$ tend to be longer/shorter in chronological order, respectively.

Let $e_{ij}(\beta )=\text{log}\;T_{ij}-\alpha_{i}-Z_{ij}^{⊤}\beta$ denote the residual of jth gap time for subject i. [1] stated that if $e_{ij}(\beta )$ lies in the observation interval of $e_{ik}(\beta )$ and, symmetrically, $e_{ik}(\beta )$ lies in the observation interval of $e_{ij}(\beta )$. Then $e_{ij}(\beta )$ and $e_{ik}(\beta )$ are comparable, and the comparability constraint is given below (pp. 792):

(5) $$e_{ij}(\beta )\leq \text{log}\left(C_{i}-S_{i,jk}-T_{ij}\right)-\left(\alpha_{i}+Z_{ik}^{⊤}\beta \right),\;e_{ik}(\beta )\leq \text{log}\left(C_{i}-S_{i,jk}-T_{ik}\right)-\left(\alpha_{i}+Z_{ij}^{⊤}\beta \right).$$

Thus $e_{ij}(\beta )$ and $e_{ik}(\beta )$ constitute a strong comparable pair if they satisfy (5). Here we want to mention that (5) is a direct extension of (2) in the presence of model (4). We will use the first inequality as an example, the first inequality is equivalent to

$$\text{log}\;T_{ij}-\alpha_{i}-Z_{ij}^{⊤}\beta \leq \text{log}\left(C_{i}-S_{i,jk}-T_{ij}\right)-\left(\alpha_{i}+Z_{ik}^{⊤}\beta \right),$$

In the absence of model (4), the first inequality of the comparability is

$$T_{ij}\leq C_{i}-S_{i,jk}-T_{ij},$$

which is the same as

$$\text{log}\;T_{ij}\leq \text{log}\left(C_{i}-S_{i,jk}-T_{ij}\right),$$

Here $C_{i}-S_{i,jk}-T_{ij}$ is the observation interval of $T_{ik}$, if $T_{ij}$ is larger than $T_{ik}$, then there is no way that $T_{ij}$ and $T_{ik}$ is comparable (since the support of $T_{ij}$ must be shorter than the support of $T_{ik}$). However, under model (4), $T_{ij}$ and $T_{ik}$ cannot be compared directly. Therefore, one needs to adjust for their corresponding covariates, as a result, we need to subtract $\alpha_{i}+Z_{ij}\beta$ on the left-hand side (since it’s related to $T_{ij}$, and subtract $\alpha_{i}+Z_{ik}\beta$ on the right-hand side (since it’s related to $T_{ik}$.

### 2.2 The Weak Comparability Under the Accelerated Failure Time Model

It is easy to see that (5) is equivalent to:

(6) $$T_{ij}+T_{ij}\text{exp}\left\{{\left(Z_{ik}-Z_{ij}\right)}^{⊤}\beta \right\}\leq C_{i}-S_{i,jk},\;T_{ik}+T_{ik}\text{exp}\left\{{\left(Z_{ij}-Z_{ik}\right)}^{⊤}\beta \right\}\leq C_{i}-S_{i,jk}.$$

In the following, we will use (6) to represent the strong comparability constraint, since the nuisance parameter $\alpha_{i}$ is eliminated. Based on (6), we propose the weak comparability constraint as follows:

(7) $$T_{ij}+T_{ik}\leq C_{i}-S_{i,jk},\;T_{ij}\text{exp}\left\{{\left(Z_{ik}-Z_{ij}\right)}^{⊤}\beta \right\}+T_{ik}\text{exp}\left\{{\left(Z_{ij}-Z_{ik}\right)}^{⊤}\beta \right\}\leq C_{i}-S_{i,jk}.$$

**Fig. 1:**
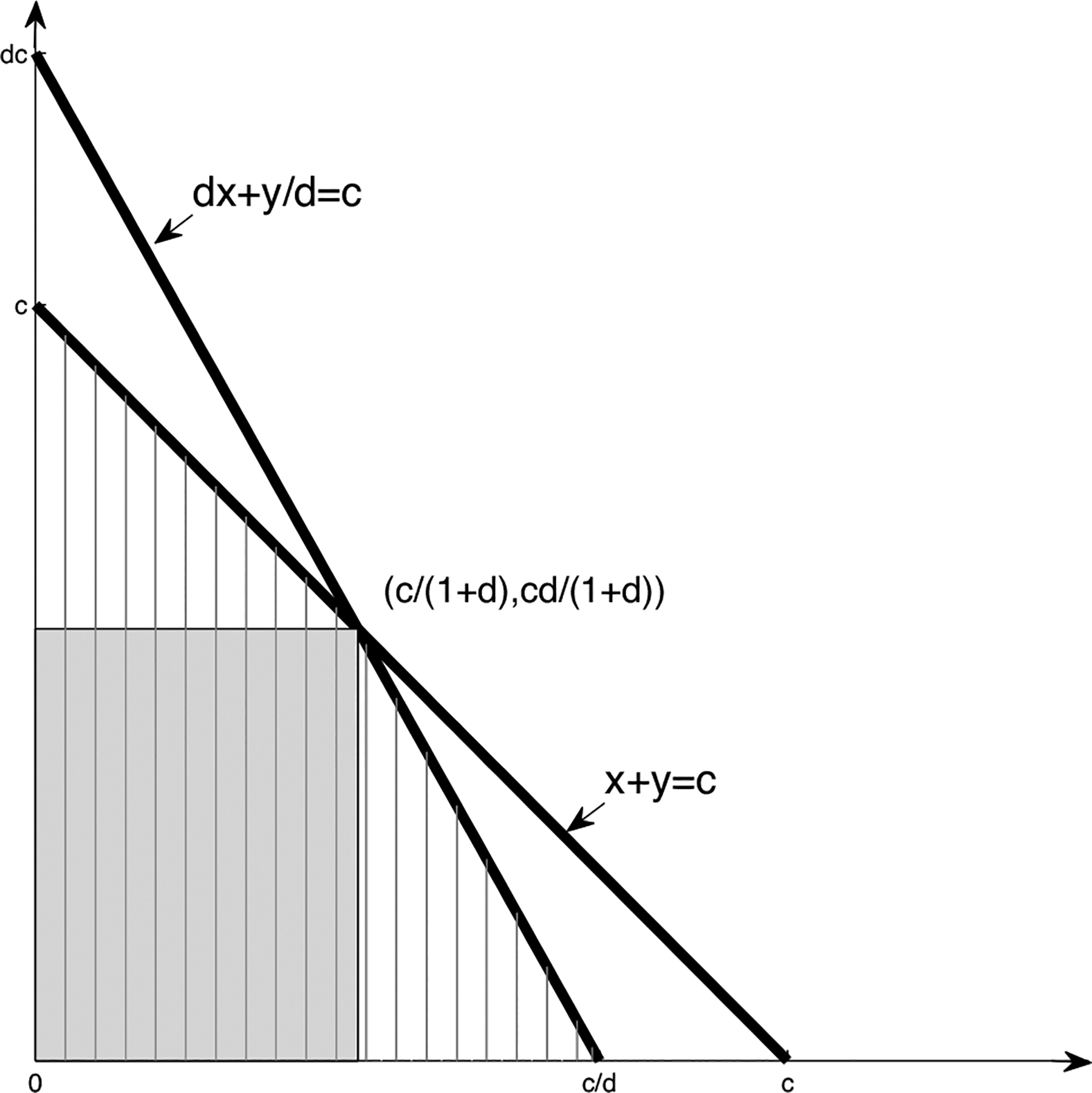
Illustration of comparability

Constraint (7) is obtained by tweaking $T_{ij}$ and $T_{ik}\text{exp}\left\{{\left(Z_{ij}-Z_{ik}\right)}^{⊤}\beta \right\}$ on the left hand side of corresponding inequalities in (6). Our intuition can be found in the geometrical shape of the constraint, which will be illustrated shortly via Fig. 1. In the following, we will first show that if $e_{ij}(\beta )$ and $e_{ik}(\beta )$ satisfy constraint (7), then they will follow the same distribution.

The assumptions that we require are as follows:

**Assumption 1** Within each subject i, the transformed gap times $\text{exp}\left(e_{ij}(\beta )\right)=T_{ij}\text{exp}\left(-\alpha_{i}-Z_{ij}^{⊤}\beta \right)$ are independently distributed given $\alpha_{i}$ and $Z_{ij}$.

**Assumption 2** Within each subject $i,C_{i}$ is conditionally independent of random intercept $\alpha_{i}$ and the random errors $\left\{e_{i1},e_{i2},\ldots \right\}$ given $Z_{i1},Z_{i2},\ldots$

Assumption 1 takes the covariate effect $Z_{ij}$ and random intercept $\alpha_{i}$ into consideration, similar assumptions can also be found in [11] and [12]. Assumption 2 means that $C_{i}$ is conditionally independent of $\left\{T_{i1},T_{i2},\ldots \right\}$ given $\left\{Z_{i1},Z_{i2},\ldots \right\}$.

**Lemma 1** *Under assumptions* 1 *and* 2*, given a fixed real value* e, *if* $e_{ij}(\beta )$ *and* $e_{ik}(\beta )$ *satisfy the strong comparability constraint* (6), *then we have*

(8) $$\text{Pr}\left\{e_{ij}(\beta )\leq e\right\}=\text{Pr}\left\{e_{ik}(\beta )\leq e\right\},$$

*if* $e_{ij}(\beta )$ *and* $e_{ik}(\beta )$ *satisfy the weak comparability constraint* (7), *then we will also have*

(9) $$\text{Pr}\left\{e_{ij}(\beta )\leq e\right\}=\text{Pr}\left\{e_{ik}(\beta )\leq e\right\}.$$

Lemma 1 shows that the constraint (7) can be used to find comparable pairs. When $e_{ij}(\beta )$ and $e_{ik}(\beta )$ satisfy the strong comparability constraint, assume the joint probability density function for $e_{ij}(\beta )$ and $e_{ik}(\beta )$ as $f_{i,jk}^{S}$, and marginal probability density functions for $e_{ij}(\beta )$ and $e_{ik}(\beta )$ as $f_{ij}^{S}$ and $f_{ik}^{S}$, respectively. When $e_{ij}(\beta )$ and $e_{ik}(\beta )$ satisfy the weak comparability constraint, then we denote the joint probability density functions and the marginal probability density functions for $e_{ij}(\beta )$ and $e_{ik}(\beta )$ as $f_{i,jk}^{W},f_{ij}^{W}$ and $f_{ik}^{W}$. It is easy to see that $f_{i,jk}^{S}=f_{ij}^{S}\times f_{ik}^{S}$, which means if $e_{ij}(\beta )$ and $e_{ik}(\beta )$ satisfy strong comparability constraint, then they are independent of each other. [1] has shown the independence of $T_{ij}$ and $T_{ik}$ in the absence of covariates, for further details, please see Sect. 2, subsection ‘Comparable $\left(t_{j},t_{k}\right)$’ in their paper. However, the same results do not hold under weak comparability. That is to say, if the comparable pairs satisfy the weak comparability constraint, we do not have $f_{i,jk}^{W}=f_{ij}^{W}\times f_{ik}^{W}$, which means the weak comparable pairs will only have the same distribution but not independent. As a result, if the comparable pairs satisfy the strong comparability constraint, then they will have the same distribution and they are also independent of each other, which means the strong comparability automatically impose an additional constraint that is not needed in estimation. However, the weak comparability constraint will not suffer from this issue. All the above results show that constraint (6) is stronger than (7).

Denote $\delta_{i,jk}^{S}(\beta )$ and $\delta_{i,jk}^{W}(\beta )$ as the indicator functions for pair $\left(T_{ij},T_{ik}\right)$ under the strong and weak comparability, respectively. If $T_{ij}$ and $T_{ik}$ satisfies (6), then $\delta_{i,jk}^{S}(\beta )=1$, and $\delta_{i,jk}^{S}(\beta )=0$ otherwise. Similarly, if $T_{ij}$ and $T_{ik}$ satisfies (7), then $\delta_{i,jk}^{W}(\beta )=1$, and $\delta_{i,jk}^{W}(\beta )=0$ otherwise. Then we can derive the unbiased estimates of parameter β by minimizing either of the following objective functions:

(10) $$M_{S}\left(\beta \right)=\sum_{i=1}^{n}\;\sum_{1\leq j<k\leq m_{i}-1}\delta_{i,jk}^{S}\left(\beta \right)\left|e_{ik}\left(\beta \right)-e_{ij}\left(\beta \right)\right|,$$

(11) $$M_{W}\left(\beta \right)=\sum_{i=1}^{n}\;\sum_{1\leq j<k\leq m_{i}-1}\delta_{i,jk}^{W}\left(\beta \right)\left|e_{ik}\left(\beta \right)-e_{ij}\left(\beta \right)\right|.$$

In the following, we show that $\delta_{i,jk}^{W}(\beta )$ will always be larger than $\delta_{i,jk}^{S}(\beta )$ for any β. To see this, let $d=\text{exp}\left\{{\left(Z_{ik}-Z_{ij}\right)}^{⊤}\beta \right\},x=T_{ij},y=T_{ik},c=C_{i}-S_{i,jk}$, then (6) becomes

(12) $$x\left(1+d\right)\leq c,\;y(1+1/d)\leq c.$$

Meanwhile, (7) is equivalent to

(13) x+y≤c,dx+y/d≤c.

In Fig. 1, the shaded area represents the strong comparability constraint, while the hatched area represents the weak comparability constraint. It is easy to see that the hatched area is bigger, which indicates $\delta_{i,jk}^{W}(\beta )\geq \delta_{i,jk}^{S}(\beta )$. As a result, (11) will utilize more gap time pairs and thus produce an estimate that has a smaller variance than the estimate obtained from (10). In addition, through simple calculation, the hatched area is $c^{2}/(1+d)$ while the shadowed area is $c^{2}d/(1+d{)}^{2}$, which shows that the hatched area is 1+1/d times larger than the shadowed area, where the d depends on $Z_{ij},Z_{ik}$ and β.

### 2.3 Asymptotic Results

Suppose (10) and (11) achieve the minimum at ${\overset{ˆ}{\beta }}_{n}^{S}$ and ${\overset{ˆ}{\beta }}_{n}^{W}$.

According to the suggestion of one of the referees, we also add some additional assumptions that are needed to establish the following theorem:

**Assumption 3** There exists a matrix $\Sigma_{0}(\beta )$ such that ${\text{lim}}_{n\to \infty }\frac{1}{n}\sum_{i=1}^{n}\text{cov}\left(\sum_{j<k}\delta_{i,jk}^{W}(\beta )\text{sgn}\left[\left(Z_{ik}-Z_{ij}\right)\left\{e_{ik}(\beta )-e_{ij}(\beta )\right\}\right]\right)=\Sigma_{0}(\beta )$.

**Assumption 4** There exists a vector μ(β) such that ${\text{lim}}_{n\to \infty }\frac{1}{n}\sum_{i=1}^{n}\sum_{j<k}\delta_{i,jk}^{W}(\beta )\text{sgn}\left[\left(Z_{ik}-Z_{ij}\right)\left\{e_{ik}(\beta )-e_{ij}(\beta )\right\}\right]=\mu (\beta )=E\left\{\sum_{j<k}\delta_{i,jk}^{W}(\beta )\text{sgn}\left[\left(Z_{ik}-Z_{ij}\right)\left\{e_{ik}(\beta )-e_{ij}(\beta )\right\}\right]\right\}$.

**Assumption 5** The probability density functions for $e_{ij}$ are continuous and bounded.

Since the two objective functions $M_{S}(\beta )$ and $M_{W}(\beta )$ have a similar form, in the following we will only present the asymptotic result for ${\overset{ˆ}{\beta }}_{n}^{W}$. Denote $\beta_{0}$ as the true value of β, then we have:

**Theorem 1** *Under Assumptions* 1 *to* 5, *the estimator* ${\overset{ˆ}{\beta }}_{n}^{W}$ *is consistent, and* $n^{1/2}\left({\overset{ˆ}{\beta }}_{n}^{W}-\beta_{0}\right)$ converges in distribution to $N\left(0,{\left\{\mu^{\prime }{\left(\beta_{0}\right)}^{⊤}\right\}}^{-1}\Sigma \left(\beta_{0}\right){\left\{\mu^{\prime }\left(\beta_{0}\right)\right\}}^{-1}\right)$, *where $\Sigma \left(\beta_{0}\right)$ is the covariance matrix of* $\sum_{j<k}\delta_{i,jk}^{W}\left(\beta_{0}\right)\text{sgn}\left[\left(Z_{ik}-Z_{ij}\right)\left\{e_{ik}\left(\beta_{0}\right)-e_{ij}\left(\beta_{0}\right)\right\}\right],\mu^{\prime }\left(\beta_{0}\right)$ *is the partial derivative of*

$$\mu (\beta )=E\left\{\sum_{j<k}\delta_{i,jk}^{W}(\beta )\text{sgn}\left[\left(Z_{ik}-Z_{ij}\right)\left\{e_{ik}(\beta )-e_{ij}(\beta )\right\}\right]\right\}$$

*at* $\beta_{0}$.

Thus $n^{1/2}\left({\overset{ˆ}{\beta }}_{n}^{W}-\beta_{0}\right)$ is asymptotic normally distributed. We need to mention that it is hard to quantify the efficiency gain for our estimator and the strong comparability due to the sandwich structure of the variance, however, as we can see from the comments in Sect. 2.1, for subject i, each $\delta_{i,jk}^{W}(\beta )$ can potentially recruit more gap time pairs than $\delta_{i,jk}^{S}(\beta )$, thus the variance for weak comparability will be smaller than the variance for strong comparability.

The estimation of the asymptotic covariance matrix maybe difficult since the numerical computations of $\mu^{\prime }\left(\beta_{0}\right)$ and $\Sigma \left(\beta_{0}\right)$ are nontrivial. Bootstrap methods can be applied in practice.

## 3 Simulation Study

In this section, we evaluate the finite sample properties of the methods developed in Sect. 2.1 via extensive simulation studies. We use the resampling approach proposed by [13] to approximate the distributions of ${\overset{ˆ}{\beta }}_{n}^{W}$ and ${\overset{ˆ}{\beta }}_{n}^{S}$. First we generate $w_{i}$ independently from the binomial distribution Bi(n,1/n), then we minimize the perturbed objective function

$${\tilde{M}}^{W}\left(\beta \right)=\sum_{i=1}^{n}w_{i}\sum_{j<k}\delta_{i,jk}^{W}\left(\beta \right)\left|e_{ik}\left(\beta \right)-e_{ij}\left(\beta \right)\right|,$$

and repeat the procedure B=200 times.

We use model (4) to generate the gap times, where we set $p=1,\alpha_{i}=1$, and assume $e_{ij}$ follows a normal distribution with mean 0 and variance 1/4. Let the true value of β equals 0 and 0.2, respectively. 500 simulated data sets were generated. We denote the number of subjects in each simulated data set as n, where we choose n=30,60,100. For ith subject in lth data set (i=1,…,n,l=1,…,500), first we generate 10 successive times, then we use a constant $C_{i}$ as the censoring time to select the first $m_{i,l}$ gap times $\left\{T_{i1},\ldots ,T_{i,m_{i,l}}\right\}$, where $\sum_{j=1}^{m_{i,l}-1}T_{ij}\leq C_{i}$ and $\sum_{j=1}^{m_{i,l}}T_{ij}>C_{i}$. We consider two trend measures in the simulation study, $Z_{ij}=j$ and $Z_{ij}=j^{1/2}$, the results for $Z_{ij}=j$ are shown in Tables 1 and 2, and the results for $Z_{ij}=j^{1/2}$ are shown in Tables 3 and 4.

For ith subject in the lth simulated data set, the observed gap times are $\left\{T_{i1},\ldots ,T_{im_{i,l}}^{+}\right\}$, denote the weak and strong comparability indicator for episodes j and $k\left(j<k\leq m_{i}-1\right)$ in subject i as $\delta_{i,jk}^{l,W}(\beta )$ and $\delta_{i,jk}^{l,S}(\beta )$, respectively. we compute the following quantities to compare the efficiency between weak and strong comparabilities:

- Mean Total Pairs: It is defined as
1500∑l=1500∑i=1nmi,l-12.
  The mean total pairs calculates the average total number of pairs and measures the maximum capacity allowed in estimation. Under this scenario, we assume that every pair is comparable, which means that for $i=1,\ldots ,n,j=1,\ldots ,m_{i},j<k\leq m_{i}-1$, we have $\delta_{i,jk}^{l,W}\left(\beta_{0}\right)=1$ and $\delta_{i,jk}^{l,S}\left(\beta_{0}\right)=1$.
- Mean Comparable Pairs: For the weak comparability, it is defined as
$$\frac{1}{500}\sum_{l=1}^{500}\sum_{i=1}^{n}\sum_{j<k\leq m_{i}-1}\delta_{i,jk}^{l,W}\left(\beta_{0}\right),$$
  while for the strong comparability, it is defined as
$$\frac{1}{500}\sum_{l=1}^{500}\sum_{i=1}^{n}\sum_{j<k\leq m_{i}-1}\delta_{i,jk}^{l,S}\left(\beta_{0}\right).$$
  The mean comparable pairs measures how much information the strong and weak comparability will utilize. The difference in mean comparable pairs for the weak and strong comparability reflects the relative efficiency of the two comparability constraints. If the mean total pairs equals to the mean comparable pairs, then every pair will be a comparable pair.

From Table 1, we can see that standard deviation for the estimates under the weak comparability constraint are smaller than the standard deviation for the estimates under the strong version. From the table, we can also see that the mean comparable pairs under weak comparability are larger than the mean comparable pairs under the strong version as well, which is already verified in a graphical way in Sect. 2.1. When the censoring time is shorter ($C_{i}=8$), the difference between the standard deviations will be larger. When the censoring time is longer (i.e. $C_{i}=9$), for each subject $i\;(i=1,\ldots ,n),m_{i}$ will be larger, thus the mean total pairs will also increase. Table 2 show similar results for $\beta_{0}=0.2$. The simulation results for $Z_{ij}=j^{1/2}$ are shown in Tables 3 and 4, and the results are similar to Tables 1 and 2. As mentioned by one of the referees, the empirical coverage probabilities in Tables 3 and 4 seem a bit low compared to the nominal level. We also conducted further simulations with larger sample sizes, and the results are shown in Tables 5 and 6. As we can see, the empirical coverage probabilities are closer to the nominal level as the sample size increases. In addition, for trend measure $Z_{ij}=j^{1/2}$, the coverage probability for strong comparability tend to be lower than the weak version under moderate or heavy censoring.

One referee comment that whether the proposed estimator performs well under other censoring mechanisms. We would like to mention that the censoring mechanisms will not affect the performance, since we only utilize the uncensored gap times and select comparable pairs (either in strong comparability or weak comparability). Based on our intuitive observation in Sect. 2.1, the comparable pairs for strong comparability will be nested within the weak comparability. So for any censoring mechanisms, the standard deviation for weak comparability will be less than the standard deviation for strong comparability. To illustrate this, we conducted a simulation study under type II censoring, where the number of gap times for each subject is the same, the results are shown in Table 7. The results also coincide with our previous findings. When the number of gap times are larger, the number of the building blocks of U statistics with subject i (i.e. $\left|e_{ik}(\beta )-e_{ij}(\beta )\right|$) will also become bigger, therefore, the standard deviation will decrease and the difference between two comparabilities will become smaller. As a further illustration, we also conducted a simulation under random censoring, the censoring time $C_{i}$ is assumed to follow an exponential distribution with mean equal to 6, the results are shown in Table 8.

Furthermore, we also conducted a simulation when the trend measure is mis-specified, here the censoring mechanism are type I and type II censoring. For type I censoring, the $C_{i}$ are set to be 8, in type II censoring, the number of gap times for each subject equals to 4, and the true trend measure is set to be $Z_{ij}=j^{1/2}$, while in the model, we assume the trend measure is $Z_{ij}=j$, the results are shown in Tables 9 and 10. From this table, we can see that both estimators perform well.

In summary, all the results indicate that the weak comparability can utilize more data, and provide a more efficient estimate than the strong comparability.

## 4 Real Data

We apply the proposed method to the HIV Prevention Trial Network 052 data [14, 15]. 1763 HIV-1-serodiscordant couples were enrolled into this study since April 2005. The study randomly assigned 1763 HIV type 1 serodiscordant couples to receive either early or delayed antiretroviral therapy treatment. 886 participants were received early therapy during enrollment, the rest 877 participants started therapy after two consecutive CD4+ counts fell below 250 cells per cubic millimeter or if an illness indicative of the acquired immunodeficiency syndrome developed. All the couples were followed up until 2015. On May 11, 2011, all the patients in both cohorts had been provided early antiretroviral therapy treatment due to an independent data and safety monitoring board of the NIH/NIAID’s recommendation. They showed a dramatically 96% risk reduction for the early antiretroviral therapy treatment arm in HIV-1 transmissions.

In this study, it is essential to remain high levels of medication adherence to recommended treatment regimes to achieve effective antiretroviral therapy treatment. Here the adherence means patients’ ability to take medications as prescribed. It is known that at least 95% of adherence is needed to achieve an effective HIV treatment [16]. In this study, the adherence was measured by pill counts, self count as well as the measurement of viral load [15]. Doctor’s counselling is recognized as one of the critical aspects that could affect adherence [17], thus all the participants in the study had received adherence counselling during each visit [14].

During the time interval between two consecutive visits, we calculate the ratio of the number of total pills that a participant has eaten versus the number of total pills that has been dispensed to this participant. We use this ratio as the measurement of adherence, and the adherence varies across different time intervals. For each participant’s visit history, we construct the recurrent event data as follows: (1) If the adherence of a participant’s first visit interval is larger than 95%, then we count the total consecutive visit days that has adherence larger than 95%, and denote the total consecutive visit episode as the high adherence episode. (2) If the adherence of a participant’s first visit interval is smaller than 95%, then we count the total consecutive visit days that has adherence smaller than 95%, and denote the consecutive visit episode as the low adherence episode. (3) Follow (1) and (2) alternatively to construct the remaining episodes, each episode serves as the gap time. (4) Define an indicator function for each episode. It equals 1 if the adherence during that episode is larger than 95%, and equals 0 otherwise. For instance, if a patient’s data is as in Table 11, then the gap times for this patient are: 14, 86 (= 28 + 58), 150 (= 60 + 90), 70, 320 (= 80 + 180 + 60), 60, with indicators equal to 0, 1, 0, 1, 0, 1. In order to maintain a high efficacy of the antiretroviral therapy treatment, an ideal pattern will be that the high adherence episodes tend to be longer, and the low adherence episodes tend to be shorter. Here we will use the proposed trend analysis method to assess the pattern of the adherence alternation. The model we consider is

(14) $$\text{log}\;T_{ij}=\alpha_{i}+Z_{ij}^{\left(1\right)}\beta_{1}+Z_{ij}^{\left(2\right)}\beta_{2}+Z_{ij}^{\left(1\right)}\times Z_{ij}^{\left(2\right)}\beta_{3}+e_{ij},$$

where $T_{ij}$ is the ith gap time for ith participant. Let $Z_{ij}^{\left(1\right)}$ denote the trend measure, here we set $Z_{ij}^{\left(1\right)}=j$ or $j^{1/2}$. Let $Z_{ij}^{\left(2\right)}$ be an indicator function, it equals to 1 if jth episode has high adherence, and equals to 0 otherwise. $\beta_{3}$ is the interaction term for the trend measure and the adherence indicator. We only focus on the 886 participants in the early antiretroviral therapy treatment arm, since the pattern of the treatment for this group is consistent from enrollment to the end of follow-up. The gap times were measured in months. Since some participants’ data were missing, we delete these participants’ data, and finally lead to 829 individuals in the analysis. The data set is analyzed under both full follow up time (without censoring) as well as an artificial censoring time (May 11, 2011). The results are shown in Table 12. All the estimates are significant in the table. From the table, we can see that ${\overset{ˆ}{\beta }}_{1}$ is positive, which indicates that the length of the higher and lower adherence episodes become longer. Positive ${\overset{ˆ}{\beta }}_{2}$ shows that the average length of the higher adherence episodes is longer than the average length of the lower adherence episodes. And negative ${\overset{ˆ}{\beta }}_{3}$ means the length of the higher adherence episodes and the lower adherence episodes change on the opposite direction – when the length of the higher adherence episodes gets longer, the length of the lower adherence episodes gets shorter, and vice versa. The results under the full follow up data and the censored data are similar. For both data, the standard deviations for ${\overset{ˆ}{\beta }}_{1}$ and ${\overset{ˆ}{\beta }}_{2}$ under weak comparability will be smaller than the standard deviations for ${\overset{ˆ}{\beta }}_{1}$ and ${\overset{ˆ}{\beta }}_{2}$ under strong comparability. Though they do not differ too much, which is due to the number of gap times is large. This is also confirmed by simulation study as well, where we can see that as the censoring time $C_{i}$ becomes longer, the difference between weak comparability and strong comparability becomes smaller.

## 5 Discussion

In this paper, we propose a new version of comparability constraint for stratified gap time under the accelerated failure time model. This constraint can be used to identify the time trend for the gap times. Compared with [1]’s comparability constraint, the proposed constraint can recruit more data pairs in estimation procedure, thus the proposed weak comparability is more efficient. The theoretical and simulation results show that our method is better than [1]’s method. We use the accelerated failure time model due to its simple interpretation. However, we also plan to extend this idea to more complicated survival models (e.g., the Cox model) in the future. While in this paper we have considered the pairwise comparison for two gap times, we will also extend the idea to compare more than two gap times.

## Figures and Tables

**Table 1:** Simulation results for $\beta_{0}=0,Z_{ij}=j$

	n	$C_{i}$	Bias×10^3^	SE×10^3^	SD×10^3^	Coverage	MCP	MTP
W	30	9	2	35	36	93.6	75.94	75.96
S	30	9	1	48	51	93.8	66.81	75.96
W	30	8	− 3	51	56	96.0	53.44	53.58
S	30	8	− 3	103	97	94.0	33.64	53.58
W	60	9	2	29	29	93.0	152.41	152.44
S	60	9	2	38	41	94.4	136.05	152.44
W	60	8	− 2	36	38	92.8	106.90	107.00
S	60	8	0	72	76	94.6	62.12	107.00
W	100	9	0	23	22	93.0	254.65	254.68
S	100	9	0	29	30	93.8	230.54	254.68
W	100	8	0	28	29	94.6	178.35	178.46
S	100	8	− 2	54	59	95.4	89.27	178.46

W weak comparability constraint, S strong comparability constraint, n number of subjects, $C_{i}$ censoring time for subject i, *Bias* estimate - $\beta_{0}$, *SE* standard error, *SD* standard deviation, *Coverage* the empirical coverage of approximate 95% confidence intervals, *MCP* mean comparable pairs, *MTP* mean total pairs

**Table 2:** Simulation results for $\beta_{0}=0.2,Z_{ij}=j$

	n	$C_{i}$	Bias×10^3^	SE×10^3^	SD×10^3^	Coverage	MCP	MTP
W	100	10	2	55	61	94.5	99.65	106.58
S	100	10	60	177	144	88.4	37.09	106.58
W	100	12	− 16	28	32	93.8	174.65	174.76
S	100	12	2	61	70	95.2	83.99	174.76
W	100	15	− 10	20	25	96.8	288.24	288.25
S	100	15	− 8	222	29	97.2	248.93	288.25
W	100	20	− 9	13	19	97.4	508.32	508.32
S	100	20	− 9	13	19	97.4	506.88	508.32

W weak comparability constraint, S strong comparability constraint, n number of subjects, $C_{i}$ censoring time for subject i, *Bias* estimate - $\beta_{0}$, *SE* standard error, *SD* standard deviation, *Coverage* the empirical coverage of approximate 95% confidence intervals, *MCP* mean comparable pairs, *MTP* mean total pairs

**Table 3:** Simulation results for $\beta_{0}=0,Z_{ij}=j^{1/2}$

	n	$C_{i}$	Bias×10^3^	SE×10^3^	SD×10^3^	Coverage	MCP	MTP
W	30	9	− 7	109	111	92.1	76.35	76.36
S	30	9	− 7	113	110	91.2	74.28	76.36
W	30	8	− 2	133	138	92.4	53.21	53.31
S	30	8	− 6	146	134	89.6	48.99	53.31
W	60	9	− 3	77	77	93.0	151.78	151.80
S	60	9	− 3	78	77	91.8	148.53	151.80
W	60	8	2	94	97	94.4	107.03	107.15
S	60	8	0	102	95	90.6	99.86	107.15
W	100	9	1	63	60	92.1	254.06	254.08
S	100	9	1	65	60	92.1	248.97	254.08
W	100	8	3	73	75	93.2	178.11	178.23
S	100	8	3	79	73	91.8	166.39	178.23

W weak comparability constraint, S strong comparability constraint, n number of subjects, $C_{i}$ censoring time for subject i, *Bias* estimate - $\beta_{0}$, *SE* standard error, *SD* standard deviation, *Coverage* the empirical coverage of approximate 95% confidence intervals, *MCP* mean comparable pairs, *MTP* mean total pairs

**Table 4:** Simulation results for $\beta_{0}=0.2,Z_{ij}=j^{1/2}$

	n	$C_{i}$	Bias×10^3^	SE×10^3^	SD×10^3^	Coverage	MCP	MTP
W	100	10	− 16	75	83	94.2	148.88	149.18
S	100	10	− 16	84	81	91.8	135.62	149.18
W	100	12	− 13	60	62	93.6	254.46	254.48
S	100	12	− 13	61	61	93.0	250.59	254.48
W	100	15	− 9	46	44	93.2	440.19	440.19
S	100	15	− 9	46	44	93.2	439.57	440.19
W	100	20	− 7	31	31	92.6	845.22	845.22
S	100	20	− 7	31	31	92.6	845.21	845.22

W weak comparability constraint, S strong comparability constraint, n number of subjects, $C_{i}$ censoring time for subject i, *Bias* estimate - $\beta_{0}$, *SE* standard error, *SD* standard deviation, *Coverage* the empirical coverage of approximate 95% confidence intervals, *MCP* mean comparable pairs, *MTP* mean total pairs

**Table 5:** Simulation results for $\beta_{0}=0,Z_{ij}=j^{1/2}$ under larger sample sizes, compared with Table 3

	n	$C_{i}$	Bias×10^3^	SE×10^3^	SD×10^3^	Coverage
W	200	9	1.7	43	43	93.5
S	200	9	0.9	46	43	95.2
W	200	8	− 4	49	53	96.8
S	200	8	− 4	51	51	94.5
W	300	9	2	33	34	94.5
S	300	9	2	35	34	94.0
W	300	8	− 2	42	42	95.0
S	300	8	− 2	45	43	93.5

W weak comparability constraint, S strong comparability constraint, n number of subjects, $C_{i}$ censoring time for subject i, *Bias* estimate - $\beta_{0}$, *SE* standard error, *SD* standard deviation, *Coverage* the empirical coverage of approximate 95% confidence intervals

**Table 6:** Simulation results for $\beta_{0}=0.2,Z_{ij}=j^{1/2}$ under larger sample sizes, compared with Tables 4

	n	$C_{i}$	Bias×10^3^	SE×10^3^	SD×10^3^	Coverage
W	200	20	− 7	21	22	94.2
S	200	20	− 7	21	22	94.2
W	300	20	− 5	17	19	94.8
S	300	20	− 5	17	19	94.8

W weak comparability constraint, S strong comparability constraint, n number of subjects, $C_{i}$ censoring time for subject i, *Bias* estimate - $\beta_{0}$, *SE* standard error, *SD* standard deviation, *Coverage* the empirical coverage of approximate 95% confidence intervals

**Table 7:** Simulation results for $\beta_{0}=0,Z_{ij}=j$ under type II censoring

	n	Number of gap times in subject i	Bias×10^3^	SE×10^3^	SD×10^3^	Coverage
W	30	3	− 20	167.48	192.12	93.5
S	30	3	− 21	186.27	195.12	92.5
W	30	4	15	96.57	102.28	95.0
S	30	4	15	96.63	102.27	94.5
W	30	5	− 1	69.08	72.61	95.2
S	30	5	− 1	69.08	72.61	95.2
W	30	6	− 5	51.41	53.01	94.8
S	30	6	− 5	51.41	53.01	94.8

W weak comparability constraint, S strong comparability constraint, n number of subjects, $C_{i}$ censoring time for subject i, *Bias* estimate - $\beta_{0}$, *SE* standard error, *SD* standard deviation, *Coverage* the empirical coverage of approximate 95% confidence intervals

**Table 8:** Simulation results for $\beta_{0}=0,Z_{ij}=j$ under random censoring

	n	Bias×10^3^	SE×10^3^	SD×10^3^	Coverage
W	30	− 3	63	66	94.8
S	30	5	70	72	94.5
W	60	− 2	45	46	95.0
S	60	− 2	50	52	94.5
W	100	− 1	35	35	95.2
S	100	− 1	35	35	95.2

W weak comparability constraint, S strong comparability constraint, n number of subjects, $C_{i}$ censoring time for subject i, *Bias* estimate - $\beta_{0}$, *SE* standard error, *SD* standard deviation, *Coverage* the empirical coverage of approximate 95% confidence intervals

**Table 9:** Simulation results under misspecification, true $\beta_{0}=0$, true $Z_{ij}=j^{\frac{1}{2}}$

	n	Number of gap times in subject i	Bias×10^3^	SE×10^3^	SD×10^3^	Coverage
W	30	4	− 2	35	38	93.5
S	30	4	− 2	35	38	93.5
W	60	4	0	28	27	90.5
S	60	4	0	28	27	90.5
W	100	4	1	20	21	92.5
S	100	4	2	20	21	92.5
W	200	4	0	14	15	94.5
S	200	4	0	14	15	94.5

In estimation $Z_{ij}=j$. Type II censoring

W weak comparability constraint, S strong comparability constraint, n number of subjects, $C_{i}$ censoring time for subject i, *Bias* estimate - $\beta_{0}$, *SE* standard error, *SD* standard deviation, *Coverage* the empirical coverage of approximate 95% confidence intervals

**Table 10:** Simulation results under misspecification, true $\beta_{0}=0$, true $Z_{ij}=j^{\frac{1}{2}}$

	n	$C_{i}$	Bias×10^3^	SE×10^3^	SD×10^3^	Coverage
W	30	8	− 11	130	133	91.5
S	30	8	− 12	143	140	90.5
W	60	8	4	87	93	93.5
S	60	8	3	92	94	92.5
W	100	8	− 1	67	68	94.5
S	100	8	− 4	74	74	92.5

In estimation $Z_{ij}=j$. Type I censoring

W weak comparability constraint, S strong comparability constraint, n number of subjects, $C_{i}$ censoring time for subject i, *Bias* estimate - $\beta_{0}$, *SE* standard error, *SD* standard deviation, *Coverage* the empirical coverage of approximate 95% confidence intervals

**Table 11:** A sample patient visit data

	1st	2nd	3rd	4th	5th	6th	7th	8th	9th	10 th
Visit time interval	14	28	58	60	90	70	80	180	60	60
Adherence indicator	0	1	1	0	0	1	0	0	0	1

Visit time interval was measured in days; adherence indicator: 1 stands for adherence larger than 95% and 0 otherwise

**Table 12:** HIV Prevention Trial Network 052 data results

			${\overset{ˆ}{\beta }}_{1}(SD)\times 10^{3}$	${\overset{ˆ}{\beta }}_{2}(SD)\times 10^{3}$	${\overset{ˆ}{\beta }}_{3}(SD)\times 10^{3}$	MCP	MTP
$Z_{ij}^{\left(1\right)}=j$	W	F	116(9)	1214(59)	− 45(5)	14844	14985
	S	F	118(9)	1032(74)	− 32(3)	14554	14985
	W	C	168(30)	905(69)	− 53(20)	4684	4711
	S	C	168(38)	981(79)	− 68(16)	4629	4711
$Z_{ij}^{\left(1\right)}=j^{1/2}$	W	F	632(37)	1128(31)	− 36(5)	14942	14985
	S	F	627(45)	969(40)	− 40(3)	14521	14985
	W	C	795(131)	967(84)	− 63(18)	4658	4711
	S	C	823(139)	899(86)	− 51(14)	4625	4711

For $Z_{ij}^{\left(1\right)}=j$, under the full data, the weak comparability utilizes 646 patients’ data, for the strong comparability, the number is 621, under censored data, the numbers are 513 and 507, respectively. For $Z_{ij}^{\left(1\right)}=j^{1/2}$, under the full data, the weak comparability utilizes 647 patients’ data, for the strong comparability, the number is 620, under censored data, the numbers are 513 and 505, respectively

W weak comparability constraint, S strong comparability constraint, F full data, C censored data, *SD* standard deviation, *MCP* mean comparable pairs, *MTP* mean total pairs

## Appendix

### Proof of Lemma 1

***Proof*** Since (8) has been proved in [1], here we will only provide a proof for (9). Assume that $e_{ij}(\beta )$ and $e_{ik}(\beta )$ satisfy constaint (7). First we consider the left part of (9):

For the first inequality of (7), notice that $T_{ij}+T_{ik}\leq C_{i}-S_{i,jk}$ is equivalent to $T_{ij}\leq C_{i}-T_{ik}-S_{i,jk}$, substitute $T_{ij}$ with $\text{exp}\left(\alpha_{i}+Z_{ij}^{⊤}\beta +e\right)$ :

$$\text{exp}\left(e_{ij}\right)\leq \left(C_{i}-S_{i,jk}-T_{ik}\right)\text{exp}\left(-\alpha_{i}-Z_{ij}^{⊤}\beta \right)\;=\left\{C_{i}-S_{i,jk}-\text{exp}\left(\alpha_{i}+Z_{ik}^{⊤}\beta +e\right)\right\}\text{exp}\left(-\alpha_{i}-Z_{ij}^{⊤}\beta \right).$$

The second inequality of (7) is equivalent to:

$$T_{ij}\text{exp}\left(-\alpha -Z_{ij}^{⊤}\beta \right)\leq \left(C_{i}-S_{i,jk}\right)\text{exp}\left(-\alpha_{i}-Z_{ik}^{⊤}\beta \right)-T_{ik}\text{exp}\left(-\alpha_{i}+Z_{ij}^{⊤}\beta -2Z_{ik}^{⊤}\beta \right).$$

Substitute $T_{ik}$ with $\text{exp}\left(\alpha_{i}+Z_{ik}^{⊤}\beta +e\right)$, we have

$$\text{exp}\left(e_{ij}\right)\leq \left(C_{i}-S_{i,jk}-T_{ij}\right)\text{exp}\left(-\alpha_{i}-Z_{ik}^{⊤}\beta \right)\;=\left\{C_{i}-S_{i,jk}-\text{exp}\left(\alpha_{i}+Z_{ij}^{⊤}\beta +e\right)\right\}\text{exp}\left(-\alpha_{i}-Z_{ik}^{⊤}\beta \right).$$

Thus

(15)
$$\text{Pr}\left\{e_{ij}(\beta )\leq e\right\}\;=\text{Pr}\left\{\text{min}\left[\text{log}\left\{C_{i}-S_{i,jk}-\text{exp}\left(\alpha_{i}+Z_{ik}^{⊤}\beta +e\right)\right\}-\alpha_{i}-Z_{ij}^{⊤}\beta ,\right.\right.\;\left.\left.\text{log}\left\{C_{i}-S_{i,jk}-\text{exp}\left(\alpha_{i}+Z_{ij}^{⊤}\beta +e\right)\right\}-\alpha_{i}-Z_{ik}^{⊤}\beta \right]\right\}.$$

Then we consider the right part of (9). Notice that $T_{ij}+T_{ik}\leq C_{i}-S_{i,jk}$ is equivalent to $T_{ik}\leq C_{i}-T_{ij}-S_{i,j}$, thus:

$$\text{exp}\left(e_{ik}\right)\leq \left(C_{i}-S_{i,jk}-T_{ij}\right)\text{exp}\left(-\alpha_{i}-Z_{ik}^{⊤}\beta \right)\;=\left\{C_{i}-S_{i,jk}-\text{exp}\left(\alpha_{i}+Z_{ij}^{⊤}\beta +e\right)\right\}\text{exp}\left(-\alpha_{i}-Z_{ik}^{⊤}\beta \right).$$

And $T_{ij}\text{exp}\left\{{\left(Z_{ik}-Z_{ij}\right)}^{⊤}\beta \right\}+T_{ik}\text{exp}\left\{{\left(Z_{ij}-Z_{ik}\right)}^{⊤}\beta \right\}\leq C_{i}-S_{i,j}$ is equivalent to

$$T_{ik}\text{exp}\left(-\alpha_{i}-Z_{ik}^{⊤}\beta \right)\leq \left(C_{i}-S_{i,j}\right)\text{exp}\left(-\alpha_{i}-Z_{ij}^{⊤}\beta \right)-T_{ij}\text{exp}\left(-\alpha_{i}+Z_{ik}^{⊤}\beta -2Z_{ij}^{⊤}\beta \right).$$

Substitute $T_{ij}$ with $\text{exp}\left(\alpha_{i}+Z_{ij}^{⊤}\beta +e\right)$, we have

$$\text{exp}\left(e_{ik}\right)\leq \left(C_{i}-S_{i,jk}-T_{ik}\right)\text{exp}\left(-\alpha_{i}-Z_{ij}^{⊤}\beta \right)\;=\left\{C_{i}-S_{i,jk}-\text{exp}\left(\alpha_{i}+Z_{ik}^{⊤}\beta +e\right)\right\}\text{exp}\left(-\alpha_{i}-Z_{ij}^{⊤}\beta \right).$$

Thus

(16)
$$\text{Pr}\left\{e_{ik}(\beta )\leq e\right\}\;=\text{Pr}\left\{\text{min}\left[\text{log}\left\{C_{i}-S_{i,jk}-\text{exp}\left(\alpha_{i}+Z_{ij}^{⊤}\beta +e\right)\right\}-\alpha_{i}-Z_{ik}^{⊤}\beta ,\right.\right.\;\left.\left.\text{log}\left\{C_{i}-S_{i,jk}-\text{exp}\left(\alpha_{i}+Z_{ik}^{⊤}\beta +e\right)\right\}-\alpha_{i}-Z_{ij}^{⊤}\beta \right]\right\}.$$

Compare equation (15) and (16), we conclude that

$$\text{Pr}\left\{e_{ij}(\beta )\leq e\right\}=\text{Pr}\left\{e_{ik}(\beta )\leq e\right\}.$$

Thus (9) is proved. □

### Proof Sketch of Theorem 1

***Proof*** To prove the asymptotic normality of the estimate ${\overset{ˆ}{\beta }}_{n}^{W}$, notice that $\delta_{i,jk}^{W}(\beta )$ in (11) is only a constraint aimed to select comparable pairs, then the derivative of (11) is:

(17)
$$M_{W}^{\prime }\left(\beta \right)=\sum_{i=1}^{n}\sum_{j<k}\delta_{i,jk}^{W}\left(\beta \right)\text{sgn}\left[{\left(Z_{ik}-Z_{ij}\right)}^{⊤}\left\{e_{ik}\left(\beta \right)-e_{ij}\left(\beta \right)\right\}\right].$$

Thus minimization of (11) is equivalent to solve $M_{W}^{\prime }(\beta )=0$. For simplicity, we also denote

$$M_{i,W}^{\prime }\left(\beta \right)=\sum_{j<k}\delta_{i,jk}^{W}\left(\beta \right)\text{sgn}\left[{\left(Z_{ik}-Z_{ij}\right)}^{⊤}\left\{e_{ik}\left(\beta \right)-e_{ij}\left(\beta \right)\right\}\right].$$

Then $M_{W}^{\prime }\left(\beta_{0}\right)=\sum_{i=1}^{n}M_{i,W}^{\prime }\left(\beta_{0}\right)$ is the sum of i.i.d. random vectors and $E\left\{M_{W}^{\prime }\left(\beta_{0}\right)\right\}=0$. Thus under the regularity conditions, $n^{-1/2}M_{W}^{\prime }\left(\beta_{0}\right)$ converges to a normal distribution. However, when consider the covariance matrix, delta method cannot being directly used since $M_{W}^{\prime }(\beta )$ is not differentiable with respect to unknown parameter β. To overcome this difficulty, we first use a smooth approximation of $M_{W}^{\prime }(\beta )$, which is $\phi (\beta )=M_{W}^{\prime }\left(\beta_{0}\right)+n\mu (\beta )$, where $\mu (\beta )=E\left[M_{i,W}^{\prime }(\beta )\right]$, we here assume that μ(β) is positive definite. As $\beta \to \beta_{0},\phi (\beta )$ is a local approximation of $M_{W}^{\prime }(\beta )$. Denote $D(\beta )=\phi (\beta )-M_{W}^{\prime }(\beta )$, then $M_{W}^{\prime }(\beta )=\phi (\beta )-D(\beta )$. By using techniques of Lemma 5 and 6 of [7], we can prove that D(β) holds a stochastic equicontinuity condition which is $D(\beta )-D\left(\beta_{0}\right)=\left\{\phi (\beta )-M_{W}^{\prime }(\beta )\right\}-\left\{\phi \left(\beta_{0}\right)-M_{W}^{\prime }\left(\beta_{0}\right)\right\}=o_{p}\left(n^{-1/2}\right)$ for $\beta -\beta_{0}=O_{p}\left(n^{-1/2}\right)$. Then we have

$$n^{-1/2}\left[M_{W}^{\prime }(\beta )-M_{W}^{\prime }\left(\beta_{0}\right)\right]\;=n^{-1/2}\left[\left\{n\mu (\beta )-n\mu \left(\beta_{0}\right)\right\}+\left\{D(\beta )-D\left(\beta_{0}\right)\right\}\right]\;=n^{1/2}\left(\beta -\beta_{0}\right)\mu^{\prime }\left(\beta_{0}\right)+o_{p}\left(1\right).$$

This completes the proof by using the functional delta method and central limit theorem, after substitute β with ${\overset{ˆ}{\beta }}_{n}^{W}$.□
